# Multimodal OCT Control for Early Histological Signs of Vulvar Lichen Sclerosus Recurrence after Systemic PDT: Pilot Study

**DOI:** 10.3390/ijms241813967

**Published:** 2023-09-12

**Authors:** Arseniy Potapov, Lev Matveev, Alexander Moiseev, Elena Sedova, Maria Loginova, Maria Karabut, Irina Kuznetsova, Viktoriya Levchenko, Elena Grebenkina, Sergey Gamayunov, Stefka Radenska-Lopovok, Marina Sirotkina, Natalia Gladkova

**Affiliations:** 1 Institute of Experimental Oncology and Biomedical Technologies, Privolzhsky Research Medical University, 603950 Nizhny Novgorod, Russia; arseniy1109@gmail.com (A.P.); natalia.gladkova@gmail.com (N.G.); 2 Institute of Applied Physics Russian Academy of Sciences, 603950 Nizhny Novgorod, Russia; lionnn52rus@mail.ru (L.M.); aleksandr.moiseev@gmail.com (A.M.); 3 Nizhny Novgorod Regional Oncologic Hospital, 603126 Nizhny Novgorod, Russia; 4 Center of Photonics, Lobachevsky State University of Nizhny Novgorod, 603022 Nizhny Novgorod, Russia; 5 Department of Obstetrics and Gynecology, Privolzhsky Research Medical University, 603950 Nizhny Novgorod, Russia; 6 N.A. Semashko Nizhny Novgorod Regional Clinical Hospital, 603126 Nizhny Novgorod, Russia; 7 Kstovo Central District Hospital, 607650 Kstovo, Russia; 8 Institute of Clinical Morphology and Digital Pathology, I.M. Sechenov First Moscow State Medical University (Sechenov University), 119991 Moscow, Russia

**Keywords:** vulva lichen sclerosus, systemic photodynamic therapy, multimodal optical coherence tomography, structural OCT, OCT angiography, OCT lymphangiography, histological signs of recurrence

## Abstract

Photodynamic therapy (PDT) is a modern treatment for severe or treatment-resistant vulvar lichen sclerosus (VLS). The chronic and recurrent nature of VLS requires control of recurrences at an early stage. In this paper, a non-invasive multimodal optical coherence tomography (OCT) method was used to control for early histological signs of VLS recurrence after systemic PDT using Photodithazine^®^. To interpret the OCT data, a histological examination was performed before PDT and 3 months after PDT. Two groups of patients were identified: with early histological signs of VLS recurrence (Group I, *n* = 5) and without histological signs of VLS recurrence (Group II, *n* = 6). We use structural OCT, OCT angiography, and OCT lymphangiography throughout 6 months after PDT to visually assess the skin components and to quantitatively assess the dermis by calculating the depth-resolved attenuation coefficient and the density of blood and lymphatic vessels. The OCT data assessment showed a statistically significant difference between the patient groups 3 months after PDT. In Group II, all the studied OCT parameters reached maximum values by the 3rd month after PDT, which indicated recovery of the skin structure. At the same time, in Group I, the values of OCT parameters did not approach the values those in Group II even after 6 months. The obtained results of multimodal OCT can be used for non-invasive control of early histological recurrence of VLS after systemic PDT and for adjusting treatment tactics in advance, without waiting for new clinical manifestations of the disease.

## 1. Introduction

Vulvar lichen sclerosus (VLS) is a chronic autoimmune inflammatory disease of the anogenital skin in women characterized by scarring of the skin and a relapsing course [1]. The classical histological pattern of the disease is pathognomonic and includes atrophy of the epidermis, homogenization of the dermis (manifested as a loss of fibrous structure) with edema of varying severity, a band-like inflammatory cell infiltration below the homogenization zone, and a sharp decrease in the number of blood and lymphatic vessels in the affected area. At an early stage of VLS, histological signs are less specific, predominantly inflammatory, and localized directly under the epidermis [2,3].

The disease is more common in premenopausal or postmenopausal women and may be less common in prepubertal girls [4]. VLS significantly reduces the quality of women’s life [5] due to the debilitating symptoms of the disease (itching, burning, and excessive sensitivity to touch), which are based on a change in the local sensitivity of the vulvar skin [6]. VLS is the main cause of structural disorders of the vulva, which lead to anatomical, sexual, and urinary dysfunction and an increased risk of developing vulvar squamous cell carcinoma. Careful and regular monitoring of women with VLS throughout life is necessary, especially in postmenopausal women [7].

The diagnosis of VLS is based on the analysis of clinical and histological signs of the disease. The diagnosis is not difficult the late stage of the disease, but the treatment of such lesions is less effective due to tissue scarring not being reversible [8]. An early diagnosis of VLS is promising, as timely treatment prevents multiple recurrences and scarring [8]. However, early VLS has non-specific symptoms, which makes diagnosis difficult.

The standard therapy for VLS involves the use of topical corticosteroids or calcineurin inhibitors, which are considered effective due to their pronounced anti-inflammatory and immunosuppressive effect [9,10]. However, in some cases, treatment does not bring the desired results. Consequently, several types of physical treatment (photodynamic therapy [11], high-intensity focused ultrasound [12], and fractional CO2 laser [13]) aimed at dermal remodeling or injective treatment (platelet-rich plasma [14] and adipose-derived stem cells [15]) aimed at skin regeneration have also been developed. Currently, no single option can be recommended for the treatment of VLS [9].

This paper discusses systemic photodynamic therapy (PDT) with Chlorin e6 derivative (Photodithazine^®^) for VLS resistant to standard therapy. This modification of PDT is commonly used in Russia [16,17,18]. In world practice, the use of systemic PDT with Photogem^®^ (hematoporphyrin derivative) [19] and Foscan^®^ (temoporfin) [20] has demonstrated high efficiency in the treatment of vulvar intraepithelial neoplasia and has ensured preservation of normal vulvar anatomy and sexual function [19]. The PDT mechanism is based on a photochemical reaction that uses a photo-activated drug (called a photosensitizer), which, when irradiated with red or near-infrared light, produces singlet oxygen that damages pathological tissue. Photodithazine^®^ (N-dimethylglucamine salt of Chlorine E6) is a second-generation photosensitizer with an absorption band in the long wavelength red region of the spectrum (λmax = 662 nm). The drug is characterized by rapid accumulation in the tissue (peak accumulation in 1.5–2.5 h after administration) and elimination rate (elimination half-life 12 h) and has low dark toxicity [21].

The most important purpose of using PDT is to achieve maximum VLS remission time without disease progression. This requires timely detection of relapse signs. The standard method of treatment control is to assess the severity of clinical signs and the vulvoscopic signs of VLS [22]. However, these methods do not allow assessing microstructural changes in the skin, indicating the development of a relapse of the disease.

In this paper, we propose to use a non-invasive method of multimodal optical coherence tomography (OCT) to control the recovery of the main components of the vulvar skin—the epidermis, dermis, blood, and lymphatic vessels—after systemic PDT in order to search for optical signs of early recurrence of VLS. Multimodal OCT is a promising method for studying surface structures of human tissues, in particular skin [23]. Firstly, the method is non-invasive, which makes it possible to repeatedly observe the tissue at short intervals at the required number of points. Secondly, the method has high resolution approaching that of the histological method. Thirdly, the method allows obtaining of a structural image in cross section (B-scan); such a section is used in the histological examination of the skin, which simplifies the interpretation of the OCT data. Fourth, the OCT signal can be quantified, which makes the OCT method objective. Multimodal OCT includes a set of studies: (1) general skin structure (structural OCT and depth-resolved attenuation coefficient calculation), (2) microcirculation of skin blood vessels (OCT angiography), and (3) lymphatic vessels (OCT lymphangiography).

Structural OCT has previously been shown to be informative both for assessing the severity [24,25] and for monitoring the effectiveness of therapy for inflammatory skin diseases [26,27]. Structural OCT imaging analysis has been used to monitor skin changes after PDT for basal cell carcinoma, squamous cell carcinoma, and actinic keratosis [28]. In our early work, OCT angiography was used to evaluate the efficacy of PDT in experimental animal tumor models [29] and in human basal cell skin carcinoma [30]. Quantitative criteria for the effectiveness of PDT were formulated according to the degree of reaction of blood vessels in the tumor and in near-tumor tissues 24 h after PDT. In this paper, for the first time, the control of the effectiveness of PDT in non-tumor pathology, in particular, VLS, was carried out using OCT angiography and OCT lymphangiography. It is known that the targets in PDT are primarily pathological cells and blood vessels [31,32]. There are no data in the literature on damage to the lymphatic vessels in PDT. Also, for the first time, the method of calculating the depth-resolved OCT attenuation coefficients of the dermis in VLS was used, which makes it possible to quantitatively and therefore objectively assess the state of the connective tissue structure and the nature of its changes after PDT.

**Figure 1:**
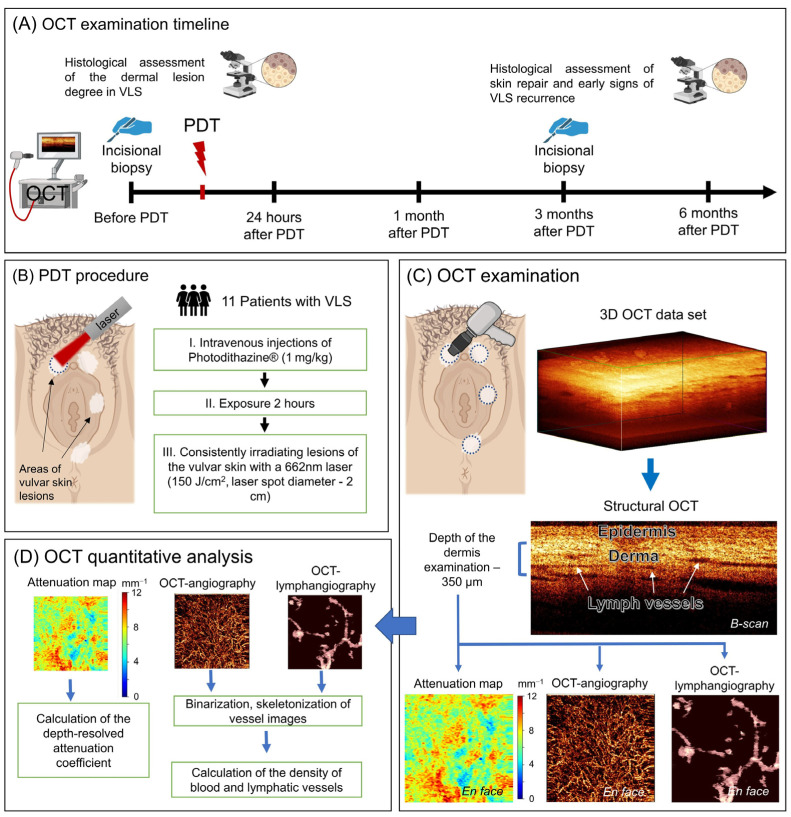
Diagram of the study design demonstrating its main steps. (**A**) Scheme of the OCT examination of vulvar skin before and after systemic PDT; (**B**) scheme of the PDT procedure; (**C**) scheme of the visual assessment of the multimodal OCT imaging; (**D**) scheme of the quantitative assessment of the multimodal OCT imaging.

Thus, the purpose of this study is visual and quantitative assessment of multimodal OCT data in order to control for early histological signs of VLS recurrence within 6 months after systemic PDT using Photodithazine^®^. In order to achieve this purpose, the multimodal OCT study was conducted before PDT to assess the degree of skin lesions 24 h, 1 month, 3 months, and 6 months after PDT to control the development of early signs of recurrence (Figure 1A). To interpret the OCT data, a parallel histological and immunohistochemical study was performed before PDT and 3 months after PDT. The points of biopsy, PDT, and OCT examinations coincided and were located in the clinically most affected areas of the vulva (Figure 1B). For visual assessment of the state of the epidermis and dermis, the multimodal OCT method was used, and structural OCT images, OCT angiographic images, and OCT lymphangiographic images, as well as color-coded attenuation coefficient maps (built based on the distribution of depth-resolved attenuation coefficient values in the dermis), were analyzed (Figure 1C). A quantitative assessment of the OCT parameters of the dermis was carried out measuring the depth-resolving attenuation coefficient and the density of blood and lymphatic vessels (Figure 1D).

## 2. Results

In VLS, all key pathological processes occur in the dermis [1]; therefore, histological changes in the dermis serve as the main criteria for the diagnosis of VLS [33]. The lesion of the dermis begins at the dermoepidermal junction (basement membrane), gradually spreading in depth [2]. Established histological changes in the epidermis are secondary and variable [34]. In this paper, histological changes in the epidermis are not analyzed in detail, so we focus on the vulvar dermis structure: fibrous connective tissue, blood, and lymphatic vessels.

### 2.1. Histological Examinations of the Skin before and 3 Months after PDT of VLS

**Figure 2:**
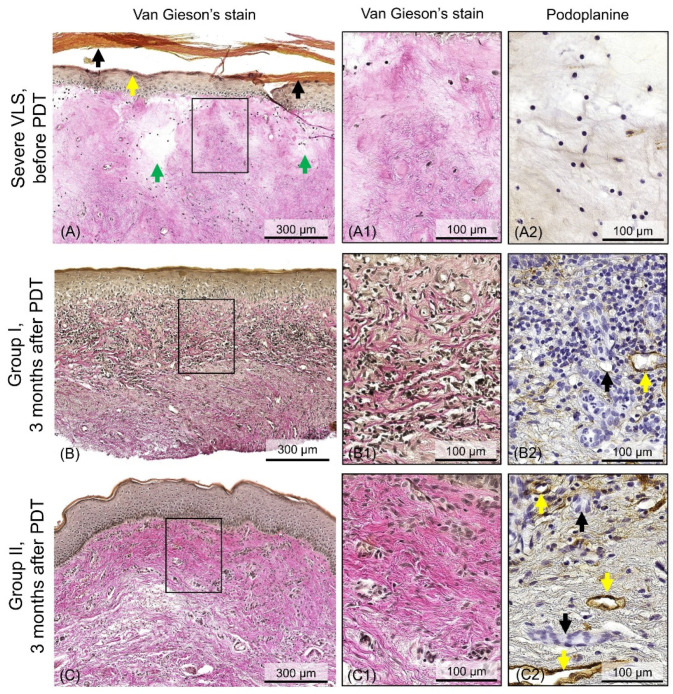
Histological and immunohistochemical study of vulvar skin samples before and 3 months after PDT of VLS. (**A**) Overview histological image of the skin in severe VLS before PDT (patient No. 3). There are signs of VLS: atrophy of the epidermis with hyperkeratosis (black arrows) and hypergranulosis (yellow arrow), homogenization of the dermis with areas of edema (green arrow) (Van Gieson’s stain, ×100); (**A1**) an enlarged area of the dermis showing absence of collagen fiber bundles—dermal homogenization (Van Gieson’s stain, ×500); (**A2**) immunohistochemical examination, the corresponding area of the dermis is demonstrated, blood and lymphatic vessels are absent (Podoplanine, ×500); (**B**) overview histological image of the skin 3 months after PDT with inflammatory signs of VLS recurrence, Group I (patient No. 8). Epidermal atrophy, the dermoepidermal junction is indistinct due to hydropic basal cell degeneration and inflammatory cell infiltration in upper dermis (Van Gieson’s stain, ×100); (**B1**) an enlarged area of the dermis, numerous inflammatory cells are located between the bundles of collagen fibers (Van Gieson’s stain, ×500); (**B2**) immunohistochemical examination, the corresponding area of the dermis is demonstrated, single blood (black arrow) and lymphatic (yellow arrow) vessels are observed among the inflammatory infiltrate (Podoplanine, ×500); (**C**) overview histological image of the skin 3 months after PDT without histological evidence of VLS recurrence, Group II (patient No. 3). The epidermis is without signs of atrophy and hyperkeratosis, the dermoepidermal junction is intact, the dermis is represented by bundles of collagen fibers, there is no inflammatory cell infiltration (Van Gieson’s stain, ×100); (**C1**) an enlarged area of the dermis demonstrates the recovery of its fibrous structure (Van Gieson’s stain, ×500); (**C2**) immunohistochemical examination, the corresponding area of the dermis is demonstrated, numerous blood (black arrows) and lymphatic (yellow arrows) vessel (Podoplanine, ×500). The enlarged areas (**A1**–**C1**,**A2**–**C2**) correspond to the selected black rectangular areas of the dermis at the depth of OCT examination of 0–350 µm.

Histological examination of the vulvar skin before PDT showed a pattern characteristic of severe VLS (according to the classification [35]). In all skin samples, epidermal atrophy was observed with signs of moderate hyperkeratosis (increase in the thickness of the keratin layer) and hypergranulosis (excessive accumulation of keratohyalin granules in cells) and total homogenization of the dermis with edema (Figure 2A). Homogenization of the dermis is the outcome of tissue destruction during VLS and is structurally a mass of extremely thin collagen fibers [35]. In the dermis, the number of blood and lymph vessels is drastically reduced (Figure 2(A1,A2)). Inflammatory cells infiltration was recorded below the area of homogenization (outside the effective depth of the OCT study).

Repeated histological examination, performed 3 months after PDT, demonstrated complete skin recovery in all patients without signs of severe VLS. However, inflammatory changes were observed in 5 patients; these changes were interpreted as signs of early histological recurrence of VLS. In the other 6 patients, no histological signs of VLS were observed. According to the results of the histological examination, patients were divided into groups: Group I, with early histological signs of VLS recurrence, and Group II, without histological signs of VLS recurrence.

Histological examination of skin samples from Group I patients after PDT demonstrated epidermal atrophy, disruption of the structure of the dermoepidermal junction and dermis due to severe inflammatory cell infiltration (Figure 2B). The inflammatory infiltrate was located between the bundles of collagen fibers in the upper dermis, at a depth of up to 350 µm (Figure 2(B1)). Blood and lymphatic vessels in the dermis are sporadic (Figure 2(B2)).

Histological examination of skin samples from Group I patients after PDT demonstrated an epidermis of normal thickness with a thin layer of keratin (Figure 2C). The dermis was represented by bundles of collagen fibers (Figure 2(C1)) and contained a large number of blood and lymphatic vessels (Figure 2(C2)), which indicates a recently completed skin recovery process after PDT. Inflammatory cell infiltration, dermal homogenization, as well as other histological signs of VLS were not detected.

### 2.2. Multimodal OCT Control for Early Histological Signs of Recurrence after PDT

OCT data are a 3D stack of 512 B-scans, which allows studying the dermis both integrally to the entire visualization depth up to 1200 µm and layer by layer at different depths (in the *en face* projection). In severe VLS, the dermis in most cases is affected to the depth of up to 1200 µm [36], which occupies the entire available scanning depth for OCT. However, changes in the dermis during the early stages of VLS develop directly under the epidermis at the depth of up to 350 µm from the basement membrane [2]. The same result is demonstrated via histological examination in the case of inflammatory changes after PDT (depth up to 350 µm—see Section 2.1). In order to perform early monitoring of inflammatory changes in the dermis, we consider the subepidermal layer of the dermis in the depth range of 0–350 µm, where the dermoepidermal junction is taken as the reference point.

In this work, we do not demonstrate multimodal OCT images of healthy vulvar skin. The multimodal OCT structure of the normal skin of the vulva is described in detail in our previous work [37].

#### 2.2.1. Visual Assessment of the Multimodal OCT Images in Group I Patients with Early Histological SIGNS of Recurrence

**Figure 3:**
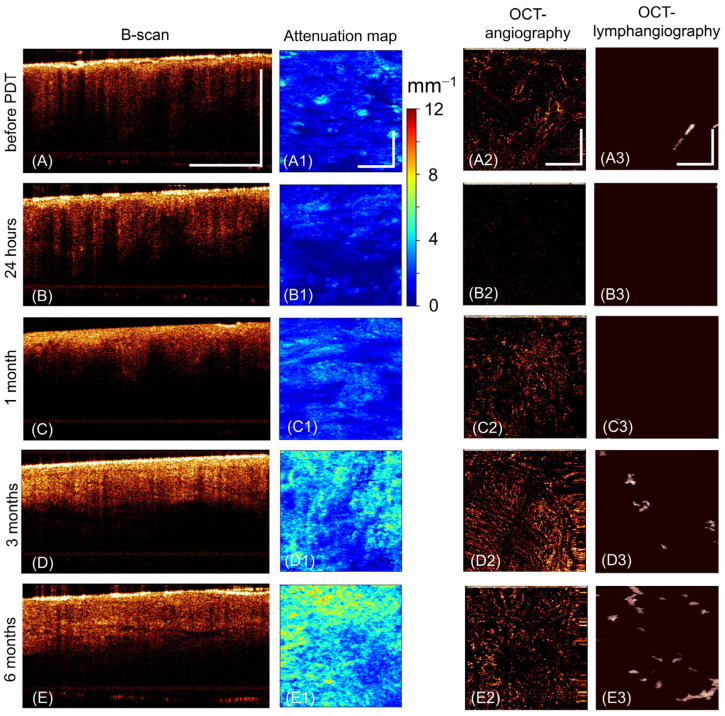
Representative OCT images of the vulvar skin of a Group I patient before PDT, 24 h after PDT, 1 month after PDT, 3 months after PDT, 6 months after PDT (patient No. 8). (**A**–**E**) Structural OCT images (B-scan); (**A1**–**E1**) dermal attenuation map (*en face*), on the right is a scale that deciphers the color code; (**A2**–**E2**) OCT angiographic image (*en face*); (**A3**–**E3**) OCT lymphangiographic images (*en face*). *En face* images are presented in the depth range of 0–350 µm from the dermoepidermal junction. The vertical and horizontal bars are 1 mm in size.

Before PDT, the vulvar skin has characteristic OCT signs of severe VLS [36]. On structural OCT images (in the B-scan projection), two layers are visualized, which are not identified throughout the entire length of the images. The first layer corresponds to the epidermis, and the second corresponds to the dermis (Figure 3A). A thin epidermis with hyperkeratosis and hypergranulosis has an intense OCT signal. The dermis exhibits a very low OCT signal that is constant throughout the depth. The low signal from the dermis is explained by the destruction of the fibrous connective tissue with the formation of vitreous homogeneous masses, which have weak backscattering properties. For the same reason, the attenuation map is dominated by the blue and dark blue color codes (Figure 3(A1)). There is a sharply reduced number of blood vessels on the OCT angiographic images (Figure 3(A2)). Lymphatic vessels, according to OCT lymphangiography (Figure 3(A3)), are absent at the studied depth in the dermis.

At 24 after *PDT*, structural OCT images become less informative (Figure 3B). The attenuation map (Figure 3(B1)) shows the dark blue color code. Vessels are almost non-existent on OCT angiographic and OCT lymphangiographic images (Figure 3(B2,B3)).

At 1 month after PDT, healing and epithelialization of PDT-treated zones show in all patients, with partially restored skin layers on structural OCT images; however, the border between the epidermis and dermis remains non-contrasting (Figure 2C). The attenuation map is dominated by the green color code (Figure 2(C1)). Single blood and lymph vessels are visualized (Figure 2(C1,C2)).

At 3 months after PDT, there is no recovery of the skin structure on the images of all OCT modalities. Layering of the tissue on the structural OCT image is disturbed due to the low contrast of the dermoepidermal junction (Figure 3D). The surface of the epidermis has a high OCT signal due to hyperkeratosis and hypergranulosis. The level of OCT signal from the dermis is lower than from the epidermis. The attenuation map shows blue and green tones (Figure 3(D1)). Single blood and lymphatic vessels are visualized (Figure 3(D2,D3)).

At 6 months after PDT, no fundamental changes are observed on OCT images, and the normal structure of the skin of the vulva is not restored (Figure 3(E,E1–E3)).

#### 2.2.2. Visual Assessment of the Multimodal OCT Images in Group II Patients without Histological Signs of Recurrence

Before PDT, 24 h and 1 month after PDT, the changes on the OCT images were fully consistent with those described for Group I (Figure 3A–C). Fundamental differences from Group I were observed only 3 and 6 months after PDT.

**Figure 4:**
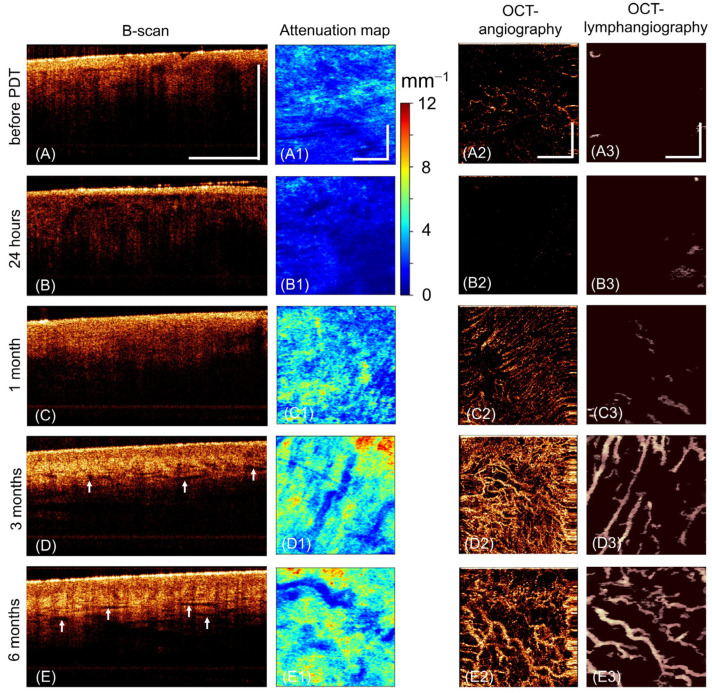
Representative OCT images of the vulvar skin of a Group I patient before PDT, 24 h after PDT, 1 month after PDT, 3 months after PDT, 6 months after PDT (patient No. 3). (**A**–**E**) Structural OCT images (B-scan), white arrows—slit-like spaces corresponding to lymphatic vessels; (**A1**–**E1**) dermal attenuation map (*en face*), on the right is a scale that deciphers the color code; (**A2**–**E2**) OCT angiographic image (*en face*); (**A3**–**E3**) OCT lymphangiographic images (*en face*)*. En face* images are presented in the depth range of 0–350 µm from the dermoepidermal junction. The vertical and horizontal bars are 1 mm in size.

At 3 months after PDT, the skin structure undergoes qualitative changes. Therefore, on structural OCT images, the recovery of the epidermal thickness and the contrast of the dermoepidermal junction are observed. The level of OCT signal from the epidermis is lower than from the dermis, which makes this image closer to the OCT image of healthy vulvar skin [37]. Slit-like formations appear in the dermis, corresponding to the lymphatic vessels (Figure 4D, white arrows). The attenuation map visualizes red and green zones (Figure 4(D1)). There is a sharp increase in the density of blood vessels and the appearance of lymphatic vessels (Figure 4(D2,D3)).

At 6 months after PDT, there are no fundamental changes in multimodal OCT images of the vulvar skin compared to 3 months (Figure 4E).

Thus, visual assessment of three types of OCT images over time demonstrated different tissue changes after PDT in patients of Groups I and II. The key period to separate patients from Groups I and II is 3 months after treatment. During this period, epithelialization of the skin has already been completed, and the structural OCT images show the recovery of two layers (corresponding to the epidermis and dermis); however, the optical properties of the layers in these groups are different. In Group II, the OCT signal from the epidermis is low, while in Group I, it is high. OCT signal from the dermis is high in Group II and low in Group I. The contrast of the layers is also different—the contrast of the dermoepidermal junction characteristic of healthy skin is restored only in Group II. The attenuation map in the upper dermis also shows differences in the condition of the dermis in the study groups, as well as recovery of blood and lymph vessels. It is important to note that these data fully correlate with our histological results obtained after 3 months of treatment and described in Section 2.1.

### 2.3. Quantitative Analysis of Multimodal OCT Parameters Control for Early Histological Signs of Recurrence after PDT

**Figure 5:**
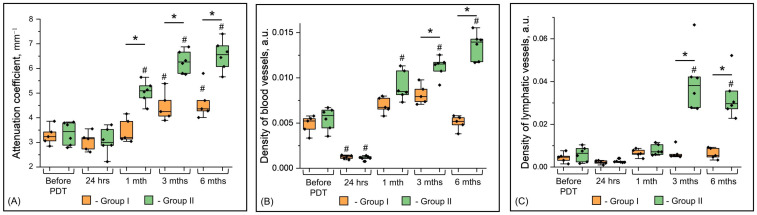
Quantitative analysis of multimodal OCT parameters of skin in patients with severe VLS (before PDT), vulva skin after PDT at different time points (24 h, 1 month, 3 months, 6 months) in patients with early histological signs of VLS recurrence (Group I) and without histological evidence of VLS recurrence (Group II). (**A**) Box-plot of depth-resolved attenuation coefficient; (**B**) box-plot of blood vessels density; (**C**) box-plot of lymphatic vessels density. Box-plot center lines represent medians. Box-plot box limits indicate upper/lower quartiles, whiskers are minimum/maximum values within the 1.5× interquartile range of the first and third quartile, and notches approximate 95% confidence intervals of the median. The dots above the box plot represent outliers. Asterisk (*)—statistical significance between unrelated patient groups (Group 1 vs. Group 2), T test with Bonferroni correction. Lattice (#)—statistical significance between the time point under study after PDT and the point before PDT, Repeated Measures ANOVA test with Bonferroni post hoc. a.u.—arbitrary units.

For the purpose of the earliest detection of OCT signs of histological recurrence of VLS after PDT, we carried out quantitative assessment of the attenuation coefficients of the OCT signal in the dermis and the density of blood and lymphatic vessels at the studied time points between patients of Groups I and II (Figure 5).

#### 2.3.1. Quantitative Analysis of the Attenuation Coefficient

The values of the attenuation coefficient of the OCT signal reflect the most general optical properties of the tissue (absorption and scattering), which depend on the density of the buds of collagen fibers, the presence of inflammatory cells, and the number of blood and lymphatic vessels in the dermis [38,39,40]. The higher the values of the attenuation coefficients are, the greater the attenuation rate is and the less transparent the tissue in the OCT range is (1310 ± 50 nm).

The attenuation coefficient OCT signal in the dermis before PDT had low values (Figure 4A). From a histological point of view, this is due to both the homogenization of the dermis (Figure 2A) and the formation of edema in severe VLS [35]. A similar effect was observed by other researchers in cerebral edema [41].

At 24 h after the PDT, the attenuation coefficient values are not statistically different from the values before the PDT.

At 1 month after PDT, there is a statistically significant difference in the attenuation coefficient values between Groups I and II (*p* = 0.003). Thus, Group II demonstrates a significant increase in attenuation coefficient values relative to the state before PDT (*p* = 0.002). In Group I, the values remain at the same low level.

At 3 and 6 months after PDT, attenuation coefficient Group II values significantly exceed Group I values (*p* = 0.0035 and *p* = 0.007, respectively).

#### 2.3.2. Quantitative Analysis of Blood Vessel Density

The most important parameter reflecting the condition of the skin of the vulva in VLS is the number of blood vessels in the dermis [42]. Initially, before PDT, blood vessel density in both groups is low (Figure 5B).

At 24 h after PDT, blood vessel density in Groups I and II decreases to almost zero (before PDT vs. 24 h, *p* = 0.023 and *p* = 0.014, respectively), which is associated with vascular thrombosis as a result of endothelial damage during the photodynamic reaction [29,31]. The result of thrombosis is necrosis of the pathological tissue (due to the special sensitivity of damaged cells to ischemia) and subsequent activation of skin regeneration.

At 1 month after PDT, blood vessel density begins to recover and exceeds the values recorded before PDT in Group II (*p* = 0.032), while in Group I it does not differ from the values before PDT (*p* = 0.64).

At 3 months after PDT, blood vessel density continues to increase in Group II, while in Group I it remains at the same level and does not differ statistically from the values before PDT (*p* = 0.17). At this time point of observation, Groups I and II differ significantly in this parameter (*p* = 0.008).

At 6 months after PDT, blood vessel density in Group II maintains an upward trend, which is statistically significantly higher than in Group I (*p* < 0.0001). Blood vessel density in Group I remains in the range that was fixed before treatment.

#### 2.3.3. Quantitative Analysis of Lymphatic Vessel Density

The study of lymphatic vessels in patients with VLS after PDT was performed by us for the first time. We demonstrate that the increase in the density and hence the number of lymphatic vessels after PDT is less than that of blood vessels (Figure 5C). In patients of Group I, enlargement of the lymphatic vessels after the procedure was not observed. Both before PDT and after PDT, lymphatic vessel density remained minimal. In patients of Group II, an increase in lymphatic vessel density was observed only 3 months after PDT (before PDT vs. 3 months, *p* = 0.034). In the 6th month after PDT, the values remained at the level of the 3rd month (before PDT vs. 6 months, *p* = 0.015).

The results of quantitative analysis of the three OCT modalities before and after PDT are presented in Table 1. The earliest OCT sign of VLS recurrence is a reduced attenuation coefficient in the dermis at 1 month after PDT. OCT angiography and lymphangiography at this time point of observation were not indicative.

Both visually and quantitatively, the key timeframe for identifying patients with early histological recurrence of VLS is 3 months after PDT. The values of all studied OCT parameters (attenuation coefficient, density of blood, and lymphatic vessels) increase significantly less in patients with VLS recurrence than in patients without histological signs of VLS recurrence. The period of 6 months does not fundamentally change this trend.

## 3. Discussion

In this paper, our research was focused on three aspects: application of multimodal OCT to control recurrence after systemic PDT and a description of early histological and corresponding OCT signs of recurrence after systemic PDT. The authors for the first time used a non-invasive high-resolution multimodal OCT method, approaching the gold standard of diagnostics regarding resolution in histological examinations, to monitor early histological signs of relapse after systemic PDT. For this purpose, an OCT device was used, which allows visualizing several modalities (tissue characteristics): depth-resolved attenuation coefficient, OCT angiography, and OCT lymphangiography. OCT lymphangiography has not been previously explored as a method of monitoring the status of the lymphatic vessels after PDT in any disease. For the first time, we have described in detail the histologically controlled use of the systemic photosensitizer Photodithazine^®^ (N-dimethylglucamine salt of Chlorine e6) (administered intravenously) for the treatment of inflammatory dermatosis (not a neoplastic process). The study describes for the first time a complete recovery of the structure of the dermis in some patients with severe VLS after PDT and early histological signs of relapse, recorded 3 months after the course of PDT in another group of patients. A detailed description of the clinical symptoms (subjective and objective signs) of the disease was not included in the objectives of our study.

In our study, the most informative parameter reflecting the recovery of vulvar tissue in the early stages of observation (1–3 months) is the depth-resolved attenuation coefficient, which was calculated using the authors’ original algorithm aimed at improving the visualization of biological tissue structures [43]. The attenuation coefficient is a complex parameter that reflects the absorption and scattering of probing radiation in the tissue [40]. The fibrous connective tissue of the dermis makes a decisive contribution to the value of the attenuation coefficient of the OCT signal (increasing the value of the coefficient), while blood vessels, lymphatic vessels, and inflammatory cell infiltration decrease the value of the attenuation coefficient. The use of an attenuation coefficient to evaluate the performance of PDT in a chemically induced squamous cell skin carcinoma model was previously described in [44], but the study did not include imaging of blood and lymph vessels. Also, the attenuation coefficient in the upper layers of the dermis was used to study two other inflammatory skin diseases—psoriasis and contact dermatitis. It was found that the attenuation coefficient in the superficial dermis is reduced in psoriasis and contact dermatitis due to the inflammatory response, which includes inflammatory cell infiltration, vasodilation, and edema, which reduces the density of collagen fiber bundles [45]. We have obtained similar results in patients with severe VLS. However, the reasons for the extremely low signal attenuation coefficient (3.32 ± 0.41 mm^−1^) in the dermis in severe VLS are different, and they are associated with the homogenization of the dermis and the loss of fibrous structure. After PDT, in patients with early histological signs of VLS recurrence, low values of the attenuation coefficient were observed at 1, 3, and 6 months after PDT, which is possibly associated with both inflammatory changes in the dermis and with the initial changes in collagen fibers. In a previous paper, we described similar changes in collagen bundles in the early stages of VLS using multiphoton microscopy [35]. It is important to note that in the described publications on the use of the attenuation coefficient to assess the effectiveness of PDT [44,45], the attenuation coefficient was calculated using log- and linear-fit methods, which visualize tissue structures to a lesser extent than the coefficient calculated using our depth-resolved method with additive noise and OCT sensitivity compensation [43].

Such an early control (1 month, 3 months) of signs of VLS recurrence is not available either for histological examination or for clinically used methods for examining vulvar skin (vulvoscopy, dermatoscopy). This is because a biopsy for VLS requires strict indications. Early biopsy after PDT (1 month) is only possible in exceptional cases [46]. Vulvoscopy and dermatoscopy are not accurate enough to visualize the structure of the dermis [47]. Thus, the great advantage of the multimodal OCT method in this area of research is its non-invasiveness and the ability to assess the state of the epidermis, dermis, blood, and lymphatic vessels repeatedly and at a sufficient depth.

In this paper, the authors first described the complete recovery of the dermal structure after systemic PDT using an intravenously administered photosensitizer in patients with severe VLS. From a histological point of view, complete tissue recovery was characterized by the absence of a zone of homogenization of the dermis and the absence of inflammatory signs of VLS. Previously published histological findings after topical PDT with 5-aminolevulinic acid (5-ALA-PDT) are inconsistent [48]. For this reason, some researchers did not record histological improvements after topical 5-ALA-PDT, and other authors stated only a decrease in the density of inflammatory cell infiltration. Furthermore, some researchers did not record histological improvements after topical 5-ALA-PDT [49,50], and other researchers stated only a decrease in the density of inflammatory cell infiltration [51]. Histological remission without specifying histological criteria is mentioned in paper [52] in two out of seventy-three patients. From the point of view of multimodal OCT, with the complete recovery of the dermal structure, we observed an increase in the values of the attenuation coefficient, the density of blood and lymphatic vessels (Figure 5, Group II). These parameters peaked at 3 and 6 months after PDT. We did not identify any scientific papers where multimodal OCT was used to evaluate the effectiveness of 5-ALA-PDT.

We attribute the complete recovery of the dermal structure observed upon histological examination to the mechanisms involved in PDT. It is known that there are three main mechanisms of action of PDT: vascular, cellular, and immune [31,32]. With topical 5-ALA-PDT, immune and cellular mechanisms are involved, which leads to a local anti-inflammatory effect. Thus, after 5-ALA-PDT, a decrease in T-lymphocytes (CD4+, CD8+) in the dermis [53] and an increase in the apoptosis index [50] in the epidermis were demonstrated. Second-generation photosensitizers are distributed more evenly in tissues due to intravenous administration. On the contrary, penetration of 5-aminolevulinic acid into the skin occurs only to the depth of 0.3–0.6 mm and depends on the thickness of the stratum corneum [54]. The application of Photodithazine^®^ also involves the vascular mechanism [55], which leads to a sharp decrease in the density of blood and lymphatic vessels to zero value 24 h after PDT (Figure 5B,C). Termination of microcirculation disturbs skin nutrition and leads to necrosis of destructive homogeneous tissue, which determines the pathological process. Subsequently, tissue is regenerated from adjacent, non-irradiated skin areas.

Another non-invasive optical method for diagnosing inflammatory skin diseases in vivo is reflective confocal microscopy (RCM). This method is clinically approved and has cellular resolution. Visualization of the skin is carried out in *en face* project (similar to the dermatoscopy field of view). The contrast of skin structures depends on their reflectivity (refractive index). Recently, RCM has been proposed as a diagnostic and control tool for standard VLS therapy in children [56]. This method has higher resolution than our multimodal OCT system and is able to visualize individual cells, which is undoubtedly important for VLS in order to determine the severity of the inflammatory infiltrate [56,57]. The main limitation of RCM is the shallow depth of the skin examination, which is 200–250 µm [57]. Such depth is sufficient to visualize the papillary dermis only in the case of atrophied epidermis and in some cases normal epidermis (the thickness of normal epidermis in the vulva is 150–200 µm [58]) and insufficient for reliable visualization of the subepidermal dermis. It should be noted that changes in the epidermis during VLS can vary and do not always lead to its atrophy. Frequent manifestations are hyperkeratosis and hypergranulosis [59], which limit optical visualization of dermis. Also, RCM does not have the ability to visualize blood and lymph vessels. It has also been demonstrated that collagen bundle changes in VLS cannot always be detected using RCM, especially if sclerosis (homogenization) is not sufficiently pronounced [60].

Unlike RCM, OCT allows one to effectively visualize the structure of the dermis to the depth of 800 µm, as well as to visualize and quantify the structure of blood and lymphatic vessels. A decrease in the number of blood and lymphatic vessels is an important marker of dermal involvement in VLS and is associated with its severity [36]. Therefore, we tend to believe that multimodal OCT is a more promising method for examining the skin in VLS, as well as other pathological skin processes accompanied by damage to the dermis. It can be assumed that the multimodal OCT method will be useful for evaluating the effectiveness of other treatments, especially if they are associated with changes in blood flow in the skin of the vulva.

Observation of the clinical manifestations (objective symptoms and subjective complaints) of VLS in Groups I and II of the studied patients was carried out for 12 months after systemic PDT. In Group I, three patients with an early clinical stage after systemic PDT had no subjective complaints, but the objective manifestations of the disease progressed. In the other two patients with a late clinical stage after systemic PDT, severe subjective manifestations (itching, burning, and dryness) persisted, and objective manifestations of the disease increased. In Group II, in three out of six patients, after systemic PDT, mild subjective symptoms (slight itching) were observed, and the progression of the objective picture of the disease did not occur.

The main limitations of this study are the small cohort of patients, absence of blinded data analysis, and the use of a locally approved photosensitizer.

The disadvantage of systemic PDT as a method of treatment is the invasiveness of the procedure. The systemic action of the photosensitizer excludes its use in patients with hepatic or renal insufficiency, and it also requires strict adherence to the light protection regime. As a result, systemic PDT requires hospitalization of the patient.

## 4. Materials and Methods

### 4.1. Patients and PDT Procedure

A total of 11 women with the diagnosis of VLS took part in the study. Patients with VLS were selected from the Department of Photodynamic Therapy and Fluorescence Diagnostics of the Nizhny Novgorod Regional Clinical Oncology Center, Russia. The diagnosis of VLS was established on the basis of clinical symptoms (according to the A. Latini classification [6]) and confirmed via histological examination. Patients were referred to PDT based on the decision of the medical council in case of non-effectiveness of standard therapy (unsuccessful use of topical corticosteroids or topical calcineurin inhibitors and no remission within three months). In patients, the clinical degree of severity of VLS was determined according to the classification [6], and the histological degree of damage to the dermis was determined according to the classification [35]. A summary of the patients included in the study is presented in Table 2. The median age of patients with VLS at the time of treatment was 61 years old (range 37–73 years old). Six women were treated with systemic PDT for the first time, and five women were treated with systemic PDT repeatedly.

The PDT procedure was performed using the clinically approved photosensitizer Photodithazine^®^ (N-dimethylglucamine salt of Chlorine e6, Veta-Grand, Moscow, Russia). Photodithazine^®^ has passed phase 3 clinical trials and is approved in the Russian Federation for systemic PDT (Registration number: ЛC-001246) [61]. This photosensitizer was included in the draft Russian guidelines for the management of VLS [62]. A patent of the Russian Federation for the treatment of VLS using Photoditazine^®^ for systemic PDT was obtained [63].

The PDT group was given intravenous administration of Photodithazine^®^ in a dose of 1 mg/kg prior to being irradiated with a 662 nm laser (LAKHTA-MILON, Moscow, Russia) at 150 J/cm^2^ at two-hour drug–light intervals. The laser spot diameter was 2 cm. The PDT procedure was performed in an operating room; patients were in the lithotomy position.

The number of irradiation sites for each patient ranged from 4 to 6 (Table 2). Skin areas with pronounced clinical signs of VLS were irradiated, and the following clinical manifestations were taken into account: skin blanching, skin atrophy, and fissures. The lesions were predominantly localized on the clitoral hood, labium minora, and posterior fourchette (Table 2). Within 24 h after the administration of the drug, patients observed a strict regime of light protection.

After the PDT procedure, all patients had a moderate pain syndrome in the area of laser irradiation, which persisted from several days to a week and was stopped by the use of non-steroidal analgesics. There were no other side effects in our group of patients.

### 4.2. Multimodal OCT Device and Data Processing

In this study, a spectral-domain multimodal OCT system “OCT 1300-E” (Biomedtech Llc, Nizhny Novgorod, Russia) with a central wavelength of 1310 nm and spectral width of 100 nm with an imaging speed of 20 kA-scans/s was used (for a more detailed description of the OCT system, see Shilyagin et al. [64]). The axial resolution of the system is 10 µm, and the lateral resolution is 15 µm. The OCT system generates 3D data set 256 pixels in depth (2 mm in air) and 512 × 512 pixels laterally (3.4 × 3.4 mm) consisting of 512 B-scans in 26 s. The OCT system is equipped with a flexible fiberoptic probe, which ends with a “pencil” type lens (length—15 cm, diameter—1 cm) and is intended for a contact examination of the tissue.

From the 3D data set, three types of images—structural OCT, OCT angiographic, and OCT lymphangiographic images—are acquired simultaneously in real time from one tissue area. To visualize blood vessels, an algorithm is used that provides compensation for uneven tissue displacement during probe or patient movement (respiratory and cardiac motions) (algorithm described in detail by Moiseev et al. [65]). The imaging method is based on temporal speckle variation as a source of contrast. For visualization of the lymphatic vessels, an algorithm based on the three-dimensional distribution of the depth-resolved attenuation coefficient is used. This approach demonstrates improved contrast and detail compared to the previously proposed approaches (algorithm described in detail by Moiseev et al. [66]). The resulting 3D vascular networks were presented in 2D images with a maximum intensity projection for blood vessels and an average intensity projection for lymphatic vessels in the 0–350 µm dermal depth range, where the dermoepidermal junction was taken as the reference point.

The software developed by our group for OCT data analysis allows us to obtain parametric images of the attenuation coefficient (color-coded maps—attenuation maps) and to obtain quantitative values of the attenuation coefficient and the density of blood and lymphatic vessels in a given depth range.

To calculate the OCT attenuation coefficient, a backscattering OCT signal was used, and the depth-resolved method proposed by K. Vermeer was applied [67]. This method was modified by our group to compensate for additive noise [68] and sensitivity of the OCT device, which depends on the scanning depth [43]. This avoids the systematic error in estimating the attenuation coefficient, which is characteristic of the K. Vermeer method. The distribution of attenuation coefficient values for each OCT data set is presented as rainbow color-coded maps “jet” in the *en face* plane. The minimum and maximum values of the color scale (blue and red, respectively) were selected taking into account the optimal contrast (the resulting value was within 0–12 mm^−1^).

A quantitative assessment of the density of blood and lymphatic vessels in the studied depth range consisted in calculating the total area of the thickness of all visualized vessels per unit area of the frame. For this purpose, the maximum intensity projection images of the blood vessels and the average intensity projection images of the lymphatic vessels were binarized, then skeletonized. Vessel lumen thickness was calculated as twice the distance between the boundary of the binary image of the vessel and its skeleton; vessels with overlapping binary boundaries and skeletons were assigned a thickness of 1 pixel. For the lateral resolution of the OCT instrument used, this corresponds to a vessel diameter of less than 15 µm.

### 4.3. Multimodal OCT Examination

A multimodal OCT study of patients with VLS was carried out in five steps: before PDT and 24 h, 1 month, 3 months, and 6 months after PDT. In total, 4 to 6 points were observed on the vulva in the area with the visually most severe skin lesion in each patient, where systemic PDT was subsequently performed (Figure 1). The localizations of the study points before and after PDT were recorded using a photo, which made it possible to accurately position the contact OCT probe at the treatment points during the follow-up assessment. According to the obtained quantitative values from 4 points of the study, the mean value was calculated.

### 4.4. Histological Examination

To conduct a histological examination, an incisional biopsy was performed twice: during the initial examination in order to make a diagnosis and 3 months after PDT to monitor early histological signs of recurrence. The first biopsy was obtained at the point of the clinically most severe tissue lesion. This area was marked with a medical skin marker and photographed. Subsequently, a systemic PDT and OCT study was carried out in this area. Each of the patient’s visits was accompanied by photofixation of the treated areas of the vulva. A repeat biopsy was obtained from the center of the irradiated area (2 cm diameter). The obtained biopsy specimens were fixed in 10% buffered neutral formalin for 48 h, and then they were dehydrated using a gradient ethanol bath, followed by xylene purification and paraffin embedding. Slices with an average thickness of 7 μ were made. Tissue sections were deparaffinized, stained, and enclosed in a synthetic mounting medium. For preliminary analysis, the samples were stained with hematoxylin and eosin (images are not presented in this work). The condition of collagen fibers was studied using Van Gieson’s stain. Verification of lymphatic vessels in the vulvar tissue was carried out via immunohistochemistry using monoclonal antibody for Podoplanin lymphatic endothelial cell markers (ab236529; Abcam, Cambridge, UK). Sections were imaged using All-in-one Type EVOS M7000 Imaging System (Thermo Fisher Scientific Inc., Waltham, MA, USA).

### 4.5. Statistical Analysis

To control early histological signs of VLS recurrence after systemic PDT, we observed consistent changes in quantitative multimodal OCT parameters in the same patients over time (before PDT, 24 h, 1 month, 3 months, and 6 months after PDT). Such observations are related and repeated, and a Repeated Measures ANOVA test with Bonferroni post hoc was applied for their analysis. Since the Repeated Measures ANOVA test requires the data to be normally distributed, the data were analyzed for normality using the Shapiro–Wilk’s test. The *p*-value was >0.15 for all groups, indicating a normal distribution of the data.

To compare the mean values of the studied quantitative OCT parameters between two groups of patients (Group I vs. Group II), an independent-samples *t*-test with Bonferroni correction was used. The Bonferroni correction was introduced by multiplying the calculated level of statistical significance by the total number of tests performed. The results were considered statistically significant if the *p*-value was <0.05.

Calculations were performed using the Statistical Package for Social Sciences 26.0 (SPSS, Chicago, IL, USA).

## 5. Conclusions

Our study successfully demonstrated the ability of multimodal OCT to control for early histological signs of relapse after systemic PDT using a second-generation intravenous administration photosensitizer in patients with severe VLS. Two histological outcomes of tissue recovery were described: the first was characterized by early histological signs of VLS recurrence; the second was most favorable without signs of VLS. It has been demonstrated that multimodal OCT including the calculation of attenuation coefficients and the calculation of the blood and lymphatic vessel density (based on OCT angiography and OCT lymphangiography) makes it possible to identify patients with histological signs of recurrence as early as 3 months after PDT. The obtained results can be used for non-invasive control over the recovery of the skin structure in patients with severe VLS after PDT already in the first three months. The persisting low values of the attenuation coefficient and the low density of blood and lymphatic vessels indicate the early development of a relapse of the disease, which will allow adjusting the treatment tactics in advance, without waiting for new clinical manifestations of the disease.

## Figures and Tables

**Table 1:** Statistical analysis of quantitative OCT parameters in patients with early histological signs of VLS recurrence (Group I) and without histological signs of VLS recurrence (Group II).

Quantitative OCT Parameters	Group I (Mean ± SD)	Group II (Mean ± SD)	*p* Value (*t*-Test with Bonferroni Correction)
	Before PDT		
Attenuation coefficient, mm^−1^	3.28 ± 0.38	3.36 ± 0.47	0.9
Density of blood vessels, a.u.	0.0048 ± 0.0009	0.0054 ± 0.0012	0.9
Density of lymphatic vessels, a.u.	0.0044 ± 0.0023	0.0060 ± 0.0034	0.9
	1 month after PDT		
Attenuation coefficient, mm^−1^	3.48 ± 0.49	5.05 ± 0.44	0.003
Density of blood vessels, a.u.	0.0069 ± 0.0009	0.0091 ± 0.0016	0.12
Density of lymphatic vessels, a.u.	0.0070 ± 0.0013	0.0071 ± 0.0024	0.9
	3 months after PDT		
Attenuation coefficient, mm^−1^	4.46 ± 0.59	6.27 ± 0.45	0.0035
Density of blood vessels, a.u.	0.0082 ± 0.0011	0.0112 ± 0.0011	0.008
Density of lymphatic vessels, a.u.	0.0066 ± 0.0029	0.04 ± 0.014	0.001
	6 months after PDT		
Attenuation coefficient, mm^−1^	4.63 ± 0.70	6.53 ± 0.62	0.007
Density of blood vessels, a.u.	0.005 ± 0.0008	0.0135 ± 0.0015	0.0001
Density of lymphatic vessels, a.u.	0.0063 ± 0.0026	0.033 ± 0.01	0.005

**Table 2:** Summary of the patients included in the study.

Patient, No.	Age, Years Old	Stages of Menopause	Histological Diagnosis and Stage [35]	Disease Duration, Years	Previous Treatment	Clinical Examination	Clinical Stage [6]	Number of Exposure Points
1	50	Menopause	VLS; Severe	15	Standard therapy *	Diffuse blanching of clitoral hood, posterior fourchette, and labium minora; asymmetric atrophic areas of the labia minora.	Early	6
2	67	Post menopause	VLS; Severe	9	Standard therapy; systemic PDT (2012)	Clitoral hood is incorporated by sclerotic tissue; complete loss of labia minora; posterior fourchette fissures.	Late	6
3	58	Post menopause	VLS; Severe	13	Standard therapy	Circumscribed blanching of clitoral hood, vaginal introitus; posterior fourchette fissures.	Early	6
4	62	Post menopause	VLS; Severe	14	Standard therapy; systemic PDT (2014)	Circumscribed blanching of clitoral hood, posterior fourchette.	Early	6
5	54	Post menopause	VLS; Severe	6	Standard therapy; systemic PDT (2016, 2017, 2018)	Diffuse blanching of clitoral hood, labium minora, vaginal introitus, and posterior fourchette.	Early	6
6	61	Post menopause	VLS; Severe	14	Standard therapy; systemic PDT (2017)	Disappearance of the clitoris; complete loss of the labia minora; atrophy of the labia majora; posterior fourchette fissures; skin lesion has a ”keyhole” pattern.	Late	6
7	61	Post menopause	VLS; Severe	8	Standard therapy; systemic PDT (2013, 2014)	Circumscribed blanching of clitoral hood, posterior fourchette; posterior fourchette fissures.	Early	4
8	67	Post menopause	VLS; Severe	9	Standard therapy	Diffuse blanching of clitoral hood, labium minora, vaginal introitus, posterior fourchette; clitoral hood is incorporated by sclerotic tissue; atrophy of the labia minora; multiple excoriations; posterior fourchette fissures.	Late	6
9	37	Peri menopause	VLS; Severe	2	Standard therapy	Diffuse blanching of clitoral hood, labium minora, vaginal introitus, posterior fourchette.	Early	5
10	73	Post menopause	VLS; Severe	8	Standard therapy	Diffuse blanching, vaginal introitus, posterior fourchette; posterior fourchette fissures.	Early	4
11	66	Post menopause	VLS; Severe	7	Standard therapy	Circumscribed blanching of clitoral hood, posterior fourchette.	Early	4

* Standard therapy includes the application of topical corticosteroids (first-line) or topical calcineurin inhibitors (second-line) combined with emollients and barrier preparations [8].

## Data Availability

The data presented in this study are available upon request from the corresponding author.
